# Strains Associated with Two 2020 Welder Anthrax Cases in the United States Belong to Separate Lineages within *Bacillus cereus sensu lato*

**DOI:** 10.3390/pathogens11080856

**Published:** 2022-07-29

**Authors:** Laura M. Carroll, Chung K. Marston, Cari B. Kolton, Christopher A. Gulvik, Jay E. Gee, Zachary P. Weiner, Jasna Kovac

**Affiliations:** 1 Structural and Computational Biology Unit, EMBL, 69117 Heidelberg, Germany; 2 Centers for Disease Control and Prevention, Atlanta, GA 30329, USA; cdk5@cdc.gov (C.K.M.); fts3@cdc.gov (C.B.K.); ylo1@cdc.gov (C.A.G.); xzg4@cdc.gov (J.E.G.); xxd7@cdc.gov (Z.P.W.); 3 Department of Food Science, The Pennsylvania State University, University Park, PA 16802, USA

**Keywords:** anthrax, *Bacillus anthracis*, *Bacillus cereus*, *Bacillus tropicus*, *Bacillus cereus* biovar Anthracis, *Bacillus cereus* group, bioterrorism, taxonomy, whole-genome sequencing, phylogenetics

## Abstract

Anthrax-causing members of *Bacillus cereus sensu lato* (*s.l.*) pose a serious threat to public health. While most anthrax-causing strains resemble *B. anthracis* phenotypically, rare cases of anthrax-like illness caused by strains resembling “*B. cereus*” have been reported. Here, whole-genome sequencing was used to characterize three *B. cereus s.l.* isolates associated with two 2020 welder anthrax cases in the United States, which resembled “*B. cereus*” phenotypically. Comparison of the three genomes sequenced here to all publicly available, high-quality *B. cereus s.l.* genomes (*n* = 2890 total genomes) demonstrated that genomes associated with each case effectively belonged to separate species at the conventional 95% average nucleotide identity prokaryotic species threshold. Two PubMLST sequence type 78 (ST78) genomes affiliated with a case in Louisiana were most closely related to *B. tropicus* and possessed genes encoding the Bps exopolysaccharide capsule, as well as hemolysin BL (Hbl) and cytotoxin K (CytK). Comparatively, a ST108 genome associated with a case in Texas was most closely related to *B. anthracis*; however, like other anthrax-causing strains most closely related to *B. anthracis*, this genome did not possess Bps-, Hbl-, or CytK-encoding genes. Overall, results presented here provide insights into the evolution of anthrax-causing *B. cereus s.l.*

## 1. Introduction

*Bacillus cereus sensu lato* (*s.l.*), also known as the *B. cereus* group, is a species complex, which encompasses numerous Gram-positive, spore forming organisms, some of which are pathogenic [1,2,3,4,5]. Among the most notorious *B. cereus s.l.* pathogens is *B. anthracis* (also known as *B. mosaicus* subsp. *anthracis* biovar Anthracis) [6], an etiologic agent of anthrax and bioterrorism agent, which has been responsible for severe infections and fatalities among humans and animals around the world [7,8,9,10]. *B. anthracis* causes anthrax via two major virulence factors, both of which are encoded by genes located on plasmids: (i) the anthrax toxins and (ii) an antiphagocytic poly-γ-D-glutamic acid (polyglutamate) capsule [7]. The three anthrax toxin proteins (i) include edema factor (EF), lethal factor (LF), and protective antigen (PA); these polypeptides are encoded by the *cya, lef,* and *pagA* genes, respectively, which are located on the pXO1 plasmid [7,11,12]. The *B. anthracis* polyglutamate capsule (ii) is produced via an operon *capBCADE*, which is located on the pXO2 plasmid [7,13].

In addition to its ability to produce anthrax toxins and a polyglutamate capsule, the *B. anthracis* species, as defined by the United States Food and Drug Administration’s Bacteriological Analytical Manual (FDA BAM; referred to hereafter as the “historical *B. anthracis* lineage”), can be distinguished from “*B. cereus*” (as defined by the FDA BAM) via phenotypic characteristics, including *B. anthracis*’ lack of motility, an inability to degrade tyrosine, lack of hemolytic activity on sheep red blood cell (RBC) agar, and lysis by γ phage [1,3,14,15,16,17]. Notably, on a genomic scale, members of the historical *B. anthracis* lineage are extremely closely related, often being referred to as clonal [6,18]. For example, using the average nucleotide identity (ANI) metric of genomic similarity [3,19,20], members of the historical *B. anthracis* lineage share ≥99.9% ANI with each other [6,18]. This can be contrasted with the 95% ANI threshold that has been largely adopted by the microbiological community as a standard prokaryotic species threshold [3,18,21], as well as the 92.5% ANI species threshold that has been proposed for *B. cereus s.l.* [6].

The majority of anthrax-causing *B. cereus s.l.* strains belong to the clonal, historical *B. anthracis* lineage [3,6,9]. However, on rare occasions, anthrax-causing strains that resemble “*B. cereus*” (per the FDA BAM) have been identified [2,5,22,23,24,25,26,27,28,29,30,31,32,33,34,35,36]. Specifically, like the historical *B. anthracis* lineage, these strains produce anthrax toxins and can cause severe illness and/or death in humans and animals [2,5,22]. However, unlike the historical *B. anthracis* lineage typically associated with anthrax illness, these strains have phenotypic characteristics typically associated with “*B. cereus*” (per the FDA BAM; e.g., motility, hemolysis on sheep RBC, not lysed by γ phage) [14,15,22,25,29,37]. Notably, some anthrax-causing *B. cereus s.l.* strains with “*B. cereus*”-like phenotypic characteristics produce the polyglutamate capsule typical of *B. anthracis* via pXO2-endcoded *cap* genes [25,37], while others produce an alternative exo-polysaccharide capsule via plasmid-encoded *bpsXABCDEFGH* [2,22,26,27,32]. Additionally, some anthrax-causing *B. cereus s.l.* strains with “*B. cereus*”-like phenotypic characteristics produce a hyaluronic acid (HA) capsule via pXO1-encoded *hasABC*; while members of the historical *B. anthracis* lineage may possess HA-encoding genes, they are not functional [27].

The isolation of anthrax-causing *B. cereus s.l.* strains with “*B. cereus*”-like phenotypic characteristics is rare. However, in 2020, the U.S. Centers for Disease Control and Prevention (CDC) isolated such strains from two separate cases of anthrax-like illness among welders in the U.S. [35]. Here, we employed whole-genome sequencing (WGS) to characterize three *B. cereus s.l.* isolates associated with these two cases of anthrax-like illness. Using the three genomes sequenced here, plus all publicly available, high-quality *B. cereus s.l.* genomes (*n* = 2890 total *B. cereus s.l.* genomes; accessed on 20 March 2021), we provide insight into the evolution and population structure of anthrax-causing *B. cereus s.l.* We further discuss potential taxonomic issues related to anthrax-causing *B. cereus s.l.* strains and how potentially dangerous misinterpretations can be avoided.

## 2. Results

### 2.1. B. cereus s.l. Strains with “B. cereus”-like Phenotypic Characteristics were Responsible for Welder Anthrax Cases in Louisiana and Texas

The genomes of three *B. cereus s.l.* isolates associated with two human clinical welder anthrax cases were sequenced in this study (Table 1 and Supplementary Table S1) [35]. Anthrax toxin-encoding genes *cya, lef,* and *pagA* were detected in all three genomes sequenced here (Table 2 and Supplementary Table S1). Notably, three separate single- or multi-locus sequence typing (SLST and MLST, respectively) methods were able to differentiate the two isolates associated with Patient F’s case in Louisiana from the isolate associated with Patient G’s case in Texas (i.e., eight-group *panC* phylogenetic group assignment, PubMLST seven-gene MLST, *rpoB* allelic typing; Table 2 and Supplementary Table S1). PubMLST’s seven-gene MLST scheme for “*B. cereus*” [38,39] assigned the Louisiana and Texas isolates to sequence types (STs) ST78 and ST108, respectively (Table 2). To avoid potential taxonomic ambiguities associated with *B. cereus s.l.* species names (discussed in detail below), the three genomes sequenced here will be discussed largely within the context of their PubMLST STs, as the PubMLST framework is well-established [38,39] and likely interpretable to most readers. 

### 2.2. Anthrax-Causing B. cereus s.l. ST78 and ST108 Genomes Belong to Separate Species at Conventional Genomospecies Thresholds

Species-level taxonomic classification of *B. cereus s.l.* strains is particularly challenging, and numerous nomenclatural frameworks have been proposed for this purpose [3,15,40,49,50,51]. Thus, the three *B. cereus s.l.* genomes sequenced here underwent taxonomic classification using multiple methods (Table 2; see Section 4.4 below for details). 

**Figure 1:**
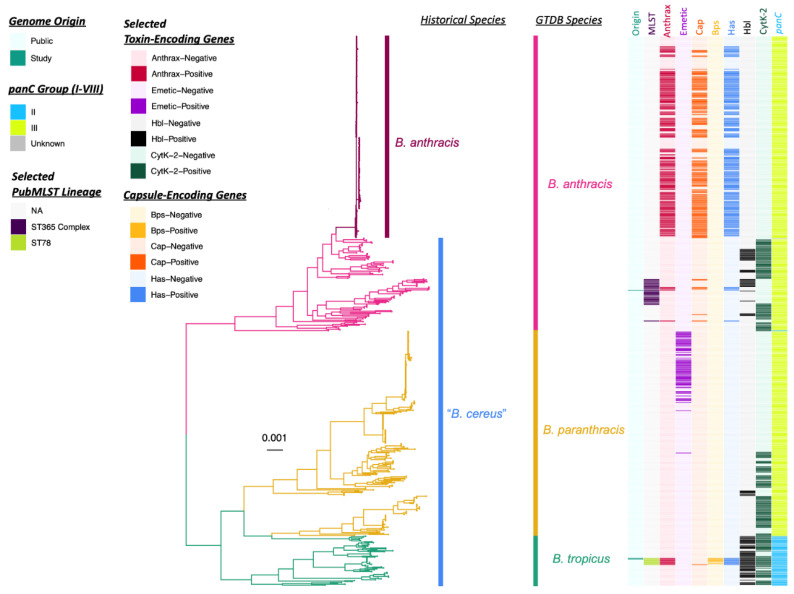
Maximum likelihood phylogeny constructed using core genes detected among 607 genomes assigned to the Genome Taxonomy Database (GTDB) *Bacillus* (*B.*) *anthracis*, *B. paranthracis*, and *B. tropicus* species, plus outgroup genome *B. cereus sensu lato* (*s.l.*) strain FSL W8-0169 (National Center for Biotechnology Information [NCBI] RefSeq Assembly Accession GCF_001583695.1; omitted for readability). Branch colors and clade labels denote GTDB species assignments or, for *B. anthracis* and Group III “*B. cereus*”, historical species assignments (per the United States Food and Drug Administration’s Bacteriological Analytical Manual [FDA BAM]). The heatmap to the right of the phylogeny denotes the following (from left to right): (i) whether a genome was sequenced in this study or publicly available (“Origin”); (ii) selected PubMLST lineages assigned using seven-gene multi-locus sequence typing, to which the three genomes sequenced in this study were assigned (“MLST”); (iii) whether a genome possessed two or more anthrax toxin-encoding genes (*cya, lef, pagA*) or not (“Anthrax”); (iv) whether a genome possessed three or more cereulide synthetase (emetic toxin)-encoding genes (*cesABCD*) or not (“Emetic”); (v) whether a genome possessed four or more polyglutamate capsule-encoding genes (*capBCADE*) or not (“Cap”); (vi) whether a genome possessed six or more Bps exopolysaccharide-encoding genes (*bpsXABCDEFGH*) or not (“Bps”); (vii) whether a genome possessed two or more hyaluronic acid capsule-encoding genes *(hasABC)* or not (“Has”); (viii) whether a genome possessed three or more hemolysin BL diarrheal enterotoxin-encoding genes (*hblABCD*) or not (“Hbl”); (ix) whether a genome possessed cytotoxin K-encoding *cytK-2* or not (“CytK-2”); (x) the *panC* Group to which each genome was assigned (using BTyper3 and an eight-group scheme; “*panC*”). The phylogeny was rooted along the outgroup genome (omitted for readability), with branch lengths reported in substitutions per site.

For one taxonomic classification approach, the three genomes sequenced here were compared to the type strain genomes of all validly published and effective *B. cereus s.l.* species (*n* = 26; accessed on 20 February 2022) using (i) average nucleotide identity (ANI) values calculated via FastANI [18], JSpeciesWS [52], and OrthoANI [53], and (ii) in silico DNA-DNA hybridization (DDH) values calculated using the Genome-to-Genome Distance Calculator (GGDC; Table 2 and Supplementary Table S1). Using all ANI- and DDH-based methods, both ST78 genomes associated with Patient F’s case in Louisiana were most closely related to the type strain genome of *B. tropicus* (Figure 1 and Table 2), a species proposed in 2017 [54]. Similarly, using the increasingly popular Genome Taxonomy Database (GTDB) framework (which itself employs ANI-based methods) [50], both ST78 genomes were assigned to GTDB’s *B. tropicus* species (Figure 1, Table 2, Supplementary Figure S1 and Table S1). However, using FastANI and OrthoANI, both ST78 genomes shared >95% ANI (widely accepted as the standard species threshold for prokaryotes) [3,18] with the type strain genomes of both *B. tropicus* and *B. paranthracis*. Conversely, using DDH, neither ST78 genome shared ≥70% DDH (also viewed as a species threshold) [45] with any *B. cereus s.l.* species type strain genome (i.e., both genomes shared 69.70% DDH with *B. tropicus*). However, the associated GGDC confidence intervals spanned the 70% species threshold (GGDC confidence interval 66.70–72.60% DDH).

Using all ANI- and DDH-based methods, the ST108 genome associated with Patient G in Texas was most closely related to *B. anthracis*, sharing >95% ANI and >70% DDH with the type strain genome (Figure 1, Table 2 and Supplementary Table S1). Congruent with this, the ST108 genome sequenced here was assigned to GTDB’s *B. anthracis* species (Figure 1, Table 2, Supplementary Figure S1 and Table S1). However, using FastANI and OrthoANI, the ST108 genome additionally shared >95% ANI with the type strain genome of *B. paranthracis*. Additionally, despite being most closely related to *B. anthracis*, the ST108 genome sequenced here did not belong to the clonal, historical *B. anthracis* lineage typically associated with anthrax illness (Figure 1). Members of the historical *B. anthracis* lineage typically responsible for anthrax illness share ≥99.9% ANI with each other [6,18], but the ST108 genome sequenced here shared 97.5% ANI with the *B. anthracis* species representative genome (via FastANI; Table 2 and Supplementary Table S1).

These results can be contrasted with taxonomic assignment using a standardized, *B. cereus s.l.*-specific genomospecies-subspecies-biovar framework (referred to hereafter as the “2020 GSB” framework; Table 2 and Supplementary Table S1). All three genomes sequenced here were assigned to the *B. mosaicus* genomospecies (i.e., a genomospecies that encompasses all *panC* Group III genomes and most *panC* Group II genomes) [40], as well as biovar Anthracis, because all genomes possessed anthrax toxin-encoding genes *cya, lef,* and *pagA* (Figure 1, Table 2, Supplementary Figure S1 and Table S1). Within the 2020 GSB framework, all three genomes can be referred to as *B. mosaicus* biovar Anthracis (full notation) or *B. anthracis* (shortened biovar notation; Figure 1, Table 2, Supplementary Figure S1 and Table S1). Notably, none of the genomes sequenced here were assigned to subspecies *anthracis* within the 2020 GSB framework, as they did not share ≥99.9% ANI with the *B. anthracis* species representative genome and thus did not belong to the clonal, historical *B. anthracis* lineage typically associated with anthrax illness (Figure 1 and Supplementary Table S1) [6].

Overall, the ST78 and ST108 genomes from the Louisiana and Texas cases, respectively, belonged to different lineages within *B. cereus s.l.*, even though all isolates possessed anthrax toxin-encoding genes and resembled “*B. cereus*” phenotypically (per the FDA BAM; Figure 1, Table 2, Supplementary Figure S1 and Table S1). The ST108 genome associated with Patient G in Texas shared 94.6% ANI and 50.5% in silico DDH with the ST78 genomes associated with Patient F’s case in Louisiana (via FastANI and GGDC, respectively; GGDC confidence interval 55.7–61.3%). Thus, each of these two major anthrax-causing lineages are discussed separately in detail below. 

### 2.3. Anthrax-Causing ST78 Genomes May Possess Genes Encoding the Bps Exopolysaccharide Capsule, as Well as Enterotoxins Hbl and CytK-2

**Figure 2:**
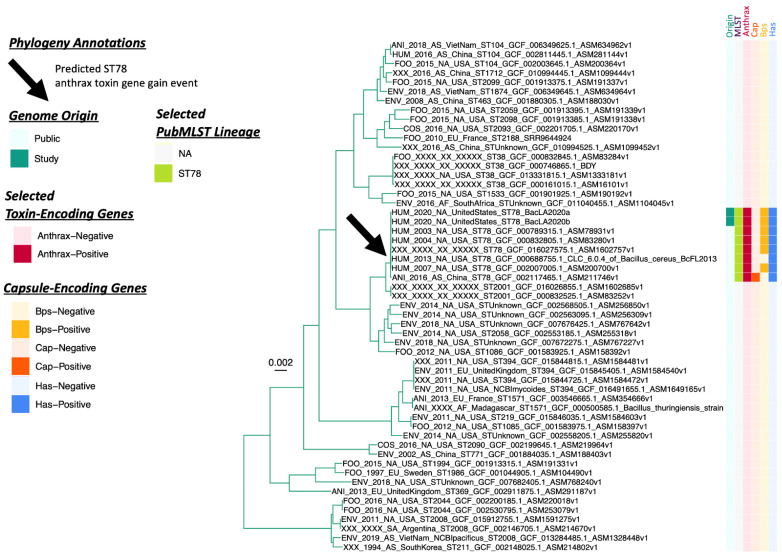
Maximum likelihood phylogeny constructed using core genes detected among 55 genomes assigned to the Genome Taxonomy Database (GTDB) *B. tropicus* species, plus GTDB *B. paranthracis* outgroup genome *B. cereus s.l.* strain AH187 (NCBI RefSeq Assembly Accession GCF_000021225.1; omitted for readability). A predicted anthrax toxin gene gain event among PubMLST Sequence Type 78 (ST78) genomes is denoted by a black arrow. The heatmap to the right of the phylogeny denotes the following (from left to right): (i) whether a genome was sequenced in this study or publicly available (“Origin”); (ii) selected PubMLST lineages assigned using seven-gene multi-locus sequence typing, to which genomes sequenced in this study were assigned (“MLST”); (iii) whether a genome possessed two or more anthrax toxin-encoding genes (*cya, lef, pagA*) or not (“Anthrax”); (iv) whether a genome possessed four or more polyglutamate capsule-encoding genes (*capBCADE*) or not (“Cap”); (v) whether a genome possessed six or more Bps exopolysaccharide-encoding genes (*bpsXABCDEFGH*) or not (“Bps”); (vi) whether a genome possessed two or more hyaluronic acid capsule-encoding genes *(hasABC)* or not (“Has”). The phylogeny was rooted along the outgroup genome (omitted for readability), with branch lengths reported in substitutions per site. For complete ancestral state reconstruction results, see Supplementary Figure S3.

The two ST78 genomes associated with Patient F’s case in Louisiana (Table 1) were nearly identical. Genomes BacLA2020a and BacLA2020b, which were derived from Patient F and a soil sample from Patient F’s worksite, respectively, shared >99.99% ANI (via FastANI, JSpeciesWS, and OrthoANI) and were identical in terms of pan-genome element presence/absence. Further, the two genomes differed by a single core SNP (identified via Snippy) in a gene annotated as sporulation kinase E. Notably, genes encoding the Bps exopolysaccharide capsule (*bpsX-H*) associated with some “atypical” anthrax-causing strains [2,27] were detected in the two ST78 genomes sequenced here (Figure 1 and Figure 2, Table 2 and Supplementary Table S1). In addition to possessing all genes encoding the Bps exopolysaccharide capsule, the two ST78 genomes sequenced here possessed hyaluronic acid (HA) capsule-encoding *hasABC*, as well as genes encoding nonhemolytic enterotoxin, hemolysin BL, and cytotoxin K variant 2 (Nhe, Hbl, and CytK-2, encoded by *nheABC, hblABCD* and *cytK-2,* respectively; Figure 1 and Figure 2, Table 2 and Supplementary Table S1). Genes encoding the poly-γ-D-glutamic acid (polyglutamate) capsule typical of anthrax-causing *B. anthracis* [2] (as defined by the FDA BAM) were not detected in either ST78 genome sequenced here (Figure 1 and Figure 2, Table 2 and Supplementary Table S1).

When compared to all publicly available *B. cereus s.l.* genomes (*n* = 2890 total genomes, accessed on 20 March 2021; Supplementary Table S2), the two ST78 genomes sequenced here shared >99.9% ANI with four *B. cereus s.l.* genomes, all of which harbored anthrax toxin-encoding genes and belonged to ST78 (Table 3 and Supplementary Table S3). While the two ST78 genomes sequenced here differed by a single core SNP, each differed from publicly available ST78 genomes by 35-1002 core SNPs (mean and median of 217.2 and 63.5 core SNPs, respectively, *n* = 8 total ST78 genomes; Supplementary Figure S2). Based on core SNP distances and phylogenetic topology, the publicly available *B. cereus s.l.* genome most closely related to the ST78 genomes sequenced here was that of *B. cereus s.l.* strain 03BB87 (National Center for Biotechnology Information [NCBI] RefSeq Assembly Accession GCF_000789315.1), a ST78 strain isolated from a fatal welder anthrax case that occurred in a human patient in Lubbock, Texas in 2003 (35–36 core SNPs relative to the ST78 genomes sequenced here; Table 3, Supplementary Figure S2 and Table S3) [37].

**Figure 3:**
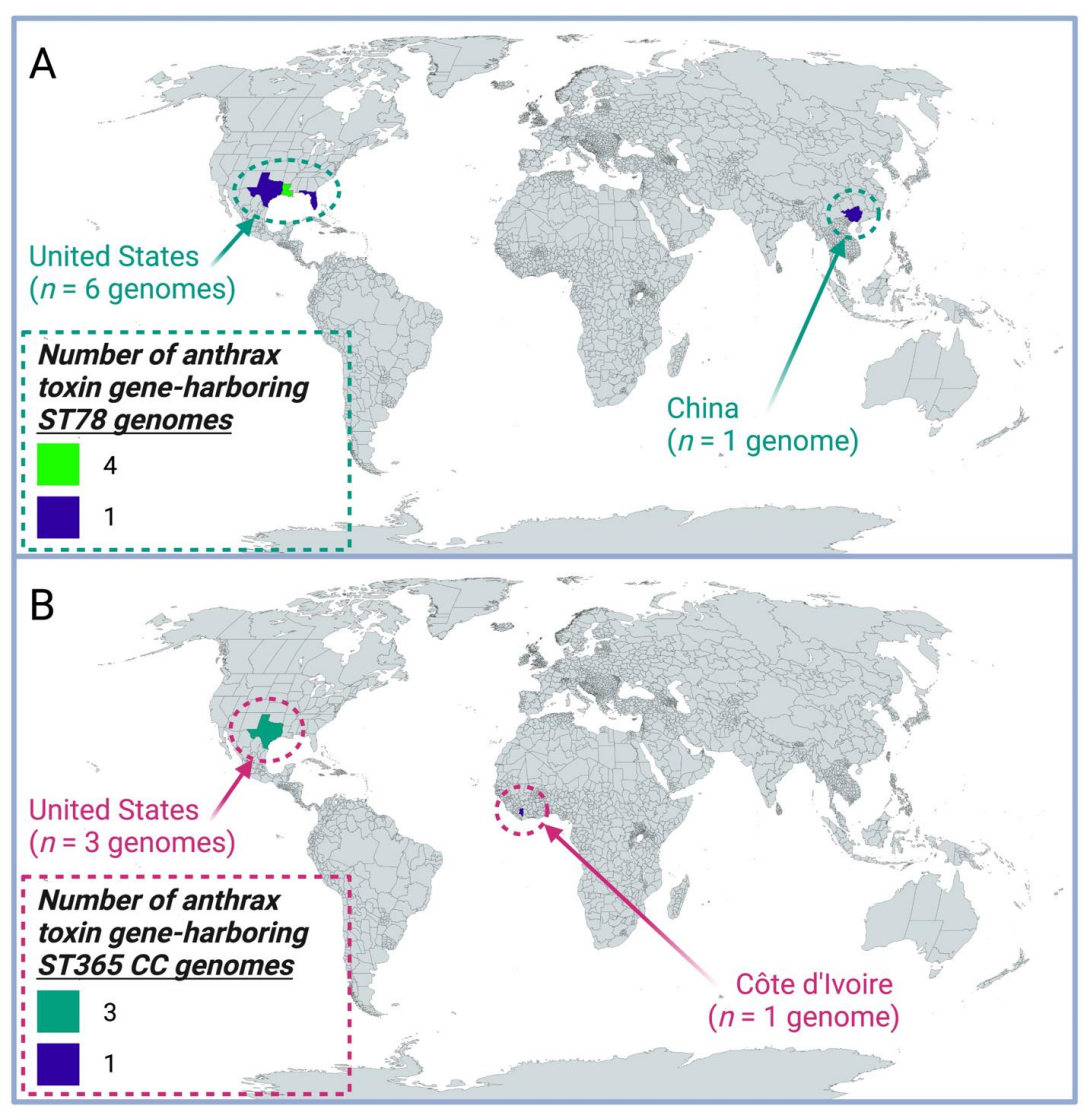
Geographic origins of anthrax toxin gene-harboring *B. cereus s.l.* genomes, which do not belong to the clonal, historical *B. anthracis* lineage. Regions are colored by the associated number of anthrax toxin gene-harboring genomes assigned to (**A**) sequence type 78 (ST78) or (**B**) the ST365 clonal complex (CC) within PubMLST. For each of (**A**) ST78 and (**B**) the ST365 CC, one anthrax toxin gene-harboring genome with an unknown origin was excluded from the map.

Like the two ST78 genomes sequenced here, all six publicly available ST78 genomes possessed anthrax toxin-encoding *cya*, *lef,* and *pagA* (8/8 total ST78 genomes, 100.0%; Figure 1, Figure 2 and Figure 3). Thus, anthrax toxin genes were predicted to have been the result of at least one acquisition event (Figure 2 and Supplementary Figure S3). All ST78 genomes additionally possessed HA capsule-encoding *hasABC*, as well as enterotoxin-encoding *nhe*, *hbl,* and *cytK-2* (8/8 total ST78 genomes, 100.0%; Figure 1, Figure 2 and Figure 3). Six ST78 genomes possessed all genes encoding the Bps exopolysaccharide capsule (6/8 total ST78 genomes, 75.0%; Figure 1, Figure 2 and Figure 3); notably, these six ST78 genomes were the only *B. cereus s.l.* genomes in which all Bps exopolysaccharide capsule-encoding genes were detected (Figure 1, Supplementary Figure S1, Supplementary Tables S1 and S2). Two ST78 genomes did not possess Bps exopolysaccharide capsule-encoding genes (Figure 1 and Figure 2). The first of these is the only ST78 genome reportedly isolated from outside of the U.S. (i.e., BC-AK, which was reportedly isolated from a kangaroo in China; Figure 3): this was the only genome not assigned to GTDB’s *B. anthracis* species that possessed polyglutamate capsule-encoding genes typical of *B. anthracis* (Figure 1 and Supplementary Figure S1). The second of the two Bps-negative ST78 genomes originated from Florida (U.S.); this genome possessed neither Bps exopolysaccharide nor polyglutamate capsule-encoding genes (Figure 1, Figure 2 and Figure 3, Table 3 and Supplementary Figure S1). 

Four of the six publicly available ST78 genomes were closed genomes; thus, the location of anthrax-associated virulence factors could be reliably evaluated. Of the four publicly available closed ST78 genomes, anthrax toxin-encoding genes were located on a plasmid, with HA capsule-encoding genes located on the same plasmid (Supplementary Table S3). However, interestingly, in one closed ST78 genome, anthrax toxin- and HA capsule-encoding genes were detected within the closed chromosome (i.e., in the anthrax-causing *B. cereus s.l.* strain 03BB87 chromosome, NCBI Nucleotide Accession NZ_CP009941.1; Supplementary Table S3). Three of four closed ST78 genomes possessed Bps exopolysaccharide capsule-encoding genes, which were located on an additional plasmid (i.e., not the same plasmid as the anthrax toxin- and HA-encoding genes; Supplementary Table S3). The sole ST78 genome that possessed polyglutamate capsule-encoding genes also harbored them on an additional plasmid (Supplementary Table S3).

Overall, within the GTDB *B. tropicus* species (i.e., the GTDB species to which all ST78 genomes were assigned), ST78 genomes were the only genomes in which anthrax toxin-, HA capsule-, Bps capsule-, and/or polyglutamate capsule-encoding genes were detected (Figure 2). Furthermore, ST78 was the only lineage not assigned to GTDB’s *B. anthracis* species, in which anthrax toxin-, HA capsule-, and/or polyglutamate capsule-encoding genes were detected (Figure 1 and Figure 2, Supplementary Figure S1, Tables S1 and S2).

**Table 3:** Publicly available anthrax toxin gene-harboring genomes, which did not belong to the clonal, historical *B. anthracis* lineage (*n* = 10) ^a^.

NCBI RefSeq Accession	Strain	Host	Geographic Location	Collection Year	GTDB Species ^b^	*panC* Group ^c^	MLST ST [CC] ^d^	Capsule ^e^
GCF_000022505.1	03BB102	Human	USA (Texas: San Antonio)	2003	*B. anthracis*	III	11 [ST-365 CC]	*cap*
GCF_000143605.1	CI	Chimpanzee (“Léo”)	Côte d′Ivoire (Taï National Park)	2002	*B. anthracis*	III	935 [ST-365 CC]	*cap*
GCF_000688755.1	BcFL2013	Human	USA (Florida)	2013	*B. tropicus*	II	78 [NA]	NA
GCF_000789315.1	03BB87	Human	USA (Texas: Lubbock)	2003	*B. tropicus*	II	78 [NA]	*bps*
GCF_000832405.1	03BB102	Human	USA (Texas: San Antonio)	2003	*B. anthracis*	III	11 [ST-365 CC]	*cap*
GCF_000832805.1	G9241	Human	USA (Louisiana)	2004	*B. tropicus*	II	78 [NA]	*bps*
GCF_002007005.1	LA2007	Human	USA (Louisiana: Galliano)	2007	*B. tropicus*	II	78 [NA]	*bps*
GCF_002117465.1	BC-AK	Kangaroo	China (Guangxi)	2016	*B. tropicus*	II	78 [NA]	*cap*
GCF_016027015.1	FDAARGOS_918	NA	NA	NA	*B. anthracis*	III	11 [ST-365 CC]	*cap*
GCF_016027575.1	FDAARGOS_897	NA	NA	NA	*B. tropicus*	II	78 [NA]	*bps*

^a^ Corresponds to all publicly available *B. cereus s.l.* genomes, which (i) were not members of *B. mosaicus* subsp. *anthracis* within the 2020 Genomospecies-Subspecies-Biovar (GSB) *B. cereus s.l.* taxonomy [6], which (ii) harbored two or more of anthrax toxin-encoding genes *cya, lef,* and *pagA*. See Supplementary Table S3 for an extended version of this table. NA, not available; ^b^ Genome Taxonomy Database (GTDB) species assigned using GTDB Release 05-RS95 (17 July 2020) and GTDB-Tk v1.3.0; ^c^ *panC* phylogenetic group (I-VIII) assigned using BTyper3 v3.2.0 [40]; ^d^ Sequence type (ST) assigned using PubMLST’s seven-gene multi-locus sequence typing (MLST) scheme for “*B. cereus*” [38,39] and BTyper3 v3.2.0 [40]. PubMLST Clonal Complex (CC) assignments are denoted in square brackets, where applicable; ^e^ Poly-γ-D-glutamic acid capsule-encoding genes *capBCADE* (*cap*) or exopolysaccharide Bps capsule-encoding genes *bpsXABCDEFGH* (*bps*) detected in each genome using BTyper3. Hyaluronic acid capsule-encoding genes (*hasABC*) were detected in all genomes. NA, not applicable, as neither *cap* nor *bps* were detected in the genome.

### 2.4. ST108 and Other Anthrax-Causing Members of the ST365 Clonal Complex Belong to B. Anthracis at Conventional Species Thresholds

**Figure 4:**
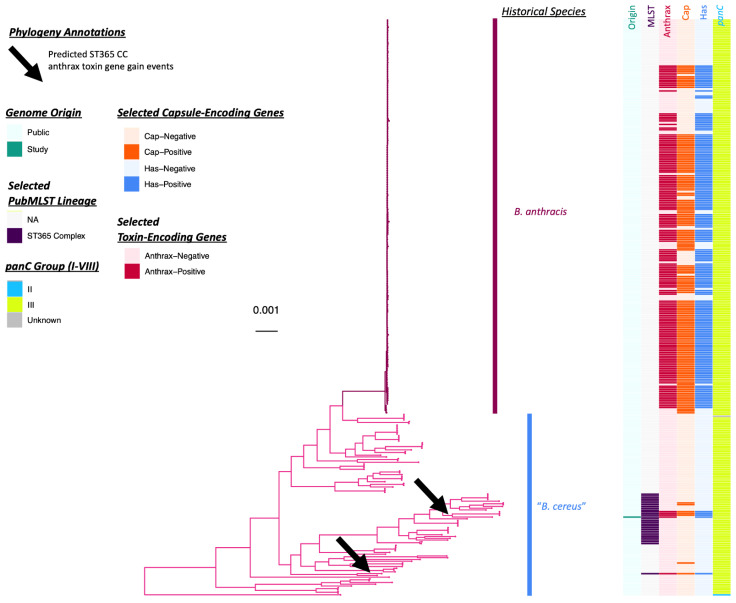
Maximum likelihood phylogeny constructed using core genes detected among 326 genomes assigned to the Genome Taxonomy Database (GTDB) *B. anthracis* species, plus GTDB *B. paranthracis* outgroup genome *B. cereus s.l.* strain AH187 (NCBI RefSeq Assembly Accession GCF_000021225.1; omitted for readability). Predicted anthrax toxin gene gain events among PubMLST Sequence Type 365 Clonal Complex (ST365 CC) genomes are denoted by black arrows. Branch colors and clade labels differentiate genomes that are members of the clonal, historical *B. anthracis* lineage (darker pink) from genomes that are not (lighter pink). The heatmap to the right of the phylogeny denotes the following (from left to right): (i) whether a genome was sequenced in this study or publicly available (“Origin”); (ii) selected PubMLST lineages assigned using seven-gene multi-locus sequence typing, to which the genomes sequenced in this study were assigned (“MLST”); (iii) whether a genome possessed two or more anthrax toxin-encoding genes (*cya, lef, pagA*) or not (“Anthrax”); (iv) whether a genome possessed four or more polyglutamate capsule-encoding genes (*capBCADE*) or not (“Cap”); (v) whether a genome possessed two or more hyaluronic acid capsule-encoding genes *(hasABC)* or not (“Has”); (vi) the *panC* Group to which each genome was assigned (using BTyper3 and an eight-group scheme; “*panC*”). The phylogeny was rooted along the outgroup genome (omitted for readability), with branch lengths reported in substitutions per site. For complete ancestral state reconstruction results, see Supplementary Figure S4.

Like the ST78 genomes associated with Patient F’s case in Louisiana, the ST108 genome associated with Patient G in Texas possessed anthrax toxin-encoding *cya, lef,* and *pagA*, as well as HA capsule-encoding *hasABC* and Nhe-encoding *nheABC* (Figure 1 and Figure 4, Table 2 and Supplementary Figure S1). However, unlike the ST78 genomes, the ST108 genome sequenced here did not possess genes encoding the Bps exopolysaccharide capsule, nor did it possess *hbl* or *cytK-2* (Figure 1 and Figure 4, Table 2 and Supplementary Figure S1). Based on ANI values calculated relative to all publicly available *B. cereus s.l.* genomes, the most closely related publicly available genome shared only 99.1% ANI with the ST108 genome sequenced here and differed by 19,425 core SNPs via FastANI and Snippy, respectively (i.e., NCBI RefSeq Assembly Accession GCF_013343075.1; Figure 1 and Figure 4 and Supplementary Figure S1).

Overall, all anthrax toxin gene-harboring *B. cereus s.l.* genomes that (i) did not belong to the clonal, historical *B. anthracis* lineage typically associated with anthrax illness and (ii) did not belong to ST78 were assigned to PubMLST’s ST365 clonal complex (CC), including the ST108 genome sequenced here (Figure 1, Figure 3 and Figure 4, Table 3, Supplementary Figure S1 and Supplementary Tables S1 and S2). Notably, all ST365 CC genomes belonged to GTDB’s *B. anthracis* species and were most closely related to the *B. anthracis* species representative genome via ANI and in silico DDH (Figure 1 and Figure 4, Table 3 and Supplementary Tables S1 and S2); however, based on user-submitted species names in NCBI, many of these strains reportedly resembled “*B. cereus*” and “*B. thuringiensis*” (Supplementary Tables S2 and S3).

Of all 30 ST365 CC genomes, five possessed anthrax toxin-encoding genes, as well as HA capsule-encoding *hasABC* (16.7% of 30 total ST365 CC genomes; Figure 1, Figure 3 and Figure 4). Notably, anthrax toxin gene-harboring members of the ST365 CC were considerably diverse: anthrax toxin gene presence within the ST365 CC was predicted to have been the result of at least two anthrax toxin gene acquisition events (Figure 4 and Supplementary Figure S4). Of the five ST365 CC genomes in which anthrax toxin-encoding genes were detected, four possessed genes encoding the polyglutamate capsule typical of anthrax-causing *B. anthracis* (80% of anthrax toxin gene-harboring ST365 CC genomes; Figure 1 and Figure 4 and Table 2 and Table 3). The only anthrax-causing ST365 CC genome that did not possess polyglutamate capsule-encoding genes was the ST108 genome sequenced here (Figure 1 and Figure 4 and Table 2). Two ST365 CC genomes, as well as one ST778 genome, possessed polyglutamate capsule-encoding genes, but did not possess anthrax toxin-encoding genes (Figure 1 and Figure 4, Supplementary Table S2). Unlike anthrax toxin gene-harboring members of ST78, anthrax toxin gene-harboring ST365 CC genomes did not possess *hbl* or *cytK-2* (Figure 1 and Supplementary Figure S1).

Of the four publicly available closed anthrax toxin gene-harboring ST365 CC genomes, anthrax toxin-, polyglutamate capsule-, and HA capsule-encoding genes were all plasmid-encoded (Supplementary Table S3). In one genome (i.e., anthrax-causing *B. cereus s.l.* strain CI), anthrax toxin- and HA capsule-encoding genes were located on one plasmid, and polyglutamate capsule-encoding genes were located on a separate plasmid (NCBI Nucleotide Accession NC_014331.1 and NC_014332.1, respectively; Supplementary Table S3). However, interestingly, in the remaining three closed genomes, all anthrax toxin-, polyglutamate capsule-, and HA capsule-encoding genes were located on a single plasmid (Supplementary Table S3).

### 2.5. Anthrax-Causing B. cereus s.l. Lineages Differ in Pan-Genome Composition

**Figure 5:**
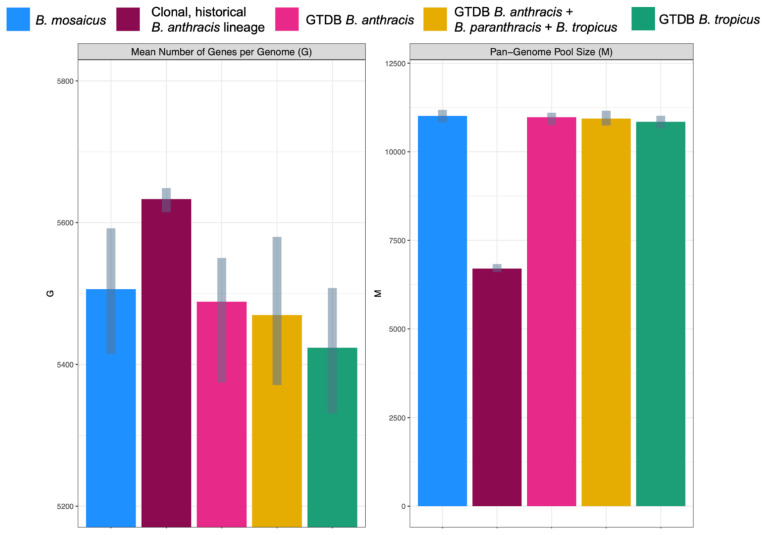
Inferred parameters for the Finitely Many Genes (FMG) model among genomes assigned to the following taxonomic units: (i) the *B. mosaicus* genomospecies within the 2020 *B. cereus s.l.* Genomospecies-Subspecies-Biovar (GSB) framework (*n* = 664 genomes)*;* (ii) the clonal, historical *B. anthracis* lineage most commonly associated with anthrax toxin production (also known as *B. mosaicus* subsp. *anthracis* within the 2020 GSB framework; *n* = 119 genomes); (iii) the Genome Taxonomy Database (GTDB) *B. anthracis* species (*n* = 180 genomes)*;* (iv) GTDB’s *B. anthracis*, *B. paranthracis*, and *B. tropicus* species (*n* = 415 genomes); (v) GTDB’s *B. tropicus* species (*n* = 46 genomes). FMG parameters were estimated using Panaroo, with gray bars denoting the 2.5 and 97.5% confidence interval bounds for each parameter (obtained using 100 bootstrap replicates).

The core- and pan-genome sizes of all *B. cereus s.l.* species (defined using GTDB or the 2020 GSB taxonomy) that contained anthrax-causing strains were compared to those of the clonal, historical *B. anthracis* lineage typically associated with anthrax illness (Figure 5). Intuitively, the historical *B. anthracis* lineage had both (i) a larger core genome and (ii) a smaller pan-genome pool size than the species defined using GTDB and the 2020 GSB taxonomy, including GTDB’s *B. anthracis* species (Figure 1 and Figure 5 and Supplementary Figure S1).

**Figure 6:**
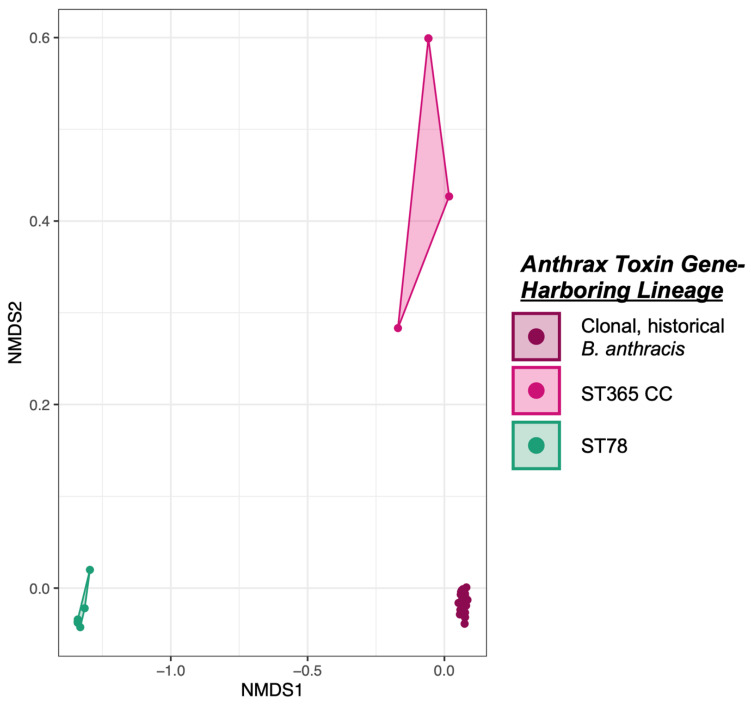
Results of non-metric multidimensional scaling (NMDS) performed using the presence and absence of pan-genome orthologous gene clusters detected among anthrax toxin gene-harboring members of (i) the clonal, historical *B. anthracis* lineage typically associated with anthrax toxin production (also known as *B. mosaicus* subsp. *anthracis* within the 2020 Genomospecies-Subspecies-Biovar [GSB] framework); (ii) the PubMLST ST365 Clonal Complex (CC;, i.e., anthrax-causing “*B. cereus*”-like genomes, which are most closely related to the *B. anthracis* species type strain genome but are not members of the 2020 GSB *B. anthracis* subspecies); (iii) PubMLST ST78 (i.e., anthrax-causing “*B. cereus*”-like genomes, which are most closely related to the *B. tropicus* species type strain genome). Points represent genomes, while shaded regions and convex hulls correspond to the anthrax toxin gene-harboring lineage to which each genome belongs. Lineages differed significantly based on pan-genome orthologous gene cluster presence/absence (Bonferroni-corrected ANOSIM and PERMANOVA *p* < 0.05).

When the pan-genomes of anthrax toxin gene-harboring *B. cereus s.l.* genomes were compared, the anthrax toxin gene-harboring (i) historical *B. anthracis*, (ii) ST78, and (iii) ST365 CC lineages each differed significantly based on pan-genome orthologous gene cluster presence/absence (Bonferroni-corrected ANOSIM and PERMANOVA *p* < 0.05; Figure 6). Among anthrax toxin gene-harboring (i) historical *B. anthracis*, (ii) ST78, and (iii) ST365 CC lineage genomes, a total of 191, 542, and 59 orthologous gene clusters were present in all anthrax toxin gene-harboring genomes within the respective lineage but absent from all other anthrax toxin gene-harboring *B. cereus s.l.* genomes, respectively (Supplementary Tables S4–S6). Likewise, a total of 39, 180, and 31 orthologous gene clusters were absent from all anthrax toxin gene-harboring genomes within the respective lineage but present in all other anthrax toxin gene-harboring *B. cereus s.l.* genomes, respectively (Supplementary Tables S7–S9). When the pan-genomes of the three aforementioned anthrax toxin gene-harboring lineages were compared to the pan-genome of *B. mosaicus* as a whole (as defined by the 2020 GSB framework; *n* = 894 total *B. mosaicus* genomes, plus outgroup *panC* Group IV genome with NCBI RefSeq Assembly Accession GCF_006094295.1), only anthrax toxin gene-harboring ST78 had orthologous gene clusters, which were exclusively present (i.e., present in all anthrax toxin gene-harboring ST78 genomes and absent from all other *B. mosaicus* genomes, plus the outgroup genome; *n =* 19 orthologous gene clusters, Supplementary Table S10). Of these 19 orthologous gene clusters exclusive to anthrax toxin gene-harboring ST78, 15 could not be assigned a function (78.9%; Supplementary Table S10). The remaining four orthologous gene clusters included (i) a deaminase with a toxin-deaminase domain (NCBI Protein Accession AIY73376.1); (ii) a response regulator (NCBI Protein Accession WP_001970204.1); (iii) a predicted restriction HNH family endonuclease (NCBI Protein Accession WP_001978396.1); (iv) a phosphorylase superfamily response regulator (NCBI Protein Accession WP_001970206.1; Supplementary Table S10).

## 3. Discussion

### 3.1. Two Distinct Lineages of Anthrax-Causing B. cereus s.l. with “B. cereus”-like Phenotypic Characteristics Are Circulating in the United States and Have the Potential to Cause Severe Anthrax-like Illness

Anthrax-causing *B. cereus s.l.* strains with a phenotypic resemblance to “*B. cereus*” (per the FDA BAM; e.g., motile, hemolytic on RBC agar) were first described in 2004, when strain G9241 was isolated from the sputum and blood of a male patient with severe inhalation anthrax [22]. In addition to its notable “*B. cereus*”-like phenotypic characteristics (per the FDA BAM), G9241 was unique in that it did not produce the pXO2-encoded polyglutamate capsule typical of *B. anthracis*; rather, it produced a hyaluronic acid (HA) capsule and alternative exopolysaccharide capsule, termed the Bps capsule [22]. Since their discovery, anthrax-causing strains with “*B. cereus*”-like phenotypic characteristics (per the FDA BAM) have been responsible for several other cases of anthrax-like disease among humans and animals [2,5,22,23,24,25,26,27,28,29,30,31,32,33,34,35,36]. Some of the strains responsible for these cases produced the *B. anthracis* polyglutamate capsule, while others produced the Bps exopolysaccharide capsule (Figure 3, Table 3 and Supplementary Table S3) [2,5,22,23,24,25,26,27,28,29,30,31,32,33,34,35,36].

Previously, we have shown that anthrax-causing *B. cereus s.l.* strains with “*B. cereus*”-like phenotypic characteristics (per the FDA BAM) belong to two separate species at the conventional 95% ANI species threshold [3,6,40]: (i) one lineage that is most closely related to the *B. anthracis* species type strain genome, but itself is distinct from the clonal, historical *B. anthracis* lineage most commonly attributed to anthrax disease, and (ii) one lineage that is most closely related to the *B. tropicus* species type strain genome. Here, we showed that both anthrax-causing “*B. cereus*”-like lineages are represented among *B. cereus s.l.* strains responsible for welder anthrax cases in the U.S. This is despite the fact that both anthrax-causing lineages queried here manifested in similar symptoms (severe pneumonia) in similar patients (male welders in their thirties) in the same year (2020) in the same U.S. region (the Gulf Coast). Both lineages of anthrax-causing *B. cereus s.l.*, which do not belong to the clonal, historical *B. anthracis* lineage, have been recently responsible for severe welder anthrax and cutaneous anthrax in the U.S. Gulf Coast region.

Using all publicly available *B. cereus s.l.* genomes, we further showed that, presently, all anthrax-causing *B. cereus s.l.* genomes that do not belong to the clonal, historical *B. anthracis* lineage belong to one of two PubMLST lineages: ST78 and the ST365 CC, which represent anthrax-causing “*B. cereus*”-like strains that are most closely related to the *B. tropicus* and *B. anthracis* species type strain genomes, respectively. Notably, these two lineages could be largely (albeit imperfectly) divided based on the type of capsule produced, as the Bps exopolysaccharide capsule first identified in strain G9241 [22] was only present in anthrax-causing ST78 strains from the U.S. (Table 2 and Table 3). However, not all anthrax-causing ST78 strains possessed genes encoding the Bps exopolysaccharide capsule. For example, ST78 strain BC-AK, which had been reportedly isolated from a kangaroo in China, possessed genes encoding the polyglutamate capsule typical of *B. anthracis* (Figure 2 and Figure 3). These results indicate that potential virulence-associated mechanistic differences between and within the anthrax-causing ST78 and ST365 CC lineages may exist, and geography may play a role in shaping these differences; however, future research is needed to elucidate this.

### 3.2. Recommendations for Effective and Unambiguous Communication of B. cereus s.l. Taxonomy for Anthrax-Causing Strains

Numerous nomenclatural frameworks exist for species-level taxonomic classification of *B. cereus s.l.* strains; recently, these frameworks have been comprehensively reviewed [3]. Currently, (i) there is no single, standardized list of *B. cereus s.l.* species accepted/used by all; (ii) there is no standardized way to assign *B. cereus s.l.* strains to species; and (iii) there is no standardized method for defining novel *B. cereus s.l.* species [3]. Furthermore, some taxonomic frameworks may not be accessible to all users. For example, researchers who do not have access to WGS cannot use some WGS-based nomenclatures [3].

Recently, we have hypothesized that the lack of standardized methods for delineating *B. cereus s.l.* species may lead to potentially dangerous misclassifications of a given strain’s virulence potential, particularly among anthrax-causing strains [3,6]. For example, as demonstrated here, the genomes of all anthrax-causing members of ST78 are most closely related to the type strain genome of *B. tropicus*, a species proposed in 2017 [54]; this is despite the fact that anthrax-causing ST78 strains have been isolated from human clinical cases for over a decade before *B. tropicus* was published as a novel species [15,22,29,31,54]. Conversely, anthrax-causing members of the ST365 CC are most closely related to *B. anthracis* on a genomic scale, despite some sharing a phenotypic resemblance to “*B. cereus*” (per the FDA BAM) [15,25,37].

To ensure that taxonomic assignments of anthrax-causing *B. cereus s.l.* strains are interpretable and unambiguous, there are several steps that readers can take, regardless of which taxonomic method they choose to employ [3]. First of all, it is recommended that readers explicitly detail the methods and protocols that were used to assign species names to strains, including the versions and access dates of all software, databases, and protocols used [3]. Secondly, readers should avoid making assumptions of virulence potential based solely on species names, particularly when the methods used to assign strains to species are unknown. For example, it is recommended to avoid treating user-assigned species names in NCBI as “ground-truth” taxonomic assignments for *B. cereus s.l.* strain genomes [3,55]. Third, readers may opt to employ a standardized SLST and/or MLST method to allow for interpretation across taxonomies [3] (e.g., the ST78 and ST108 genomes sequenced here can be referred to as such, independent of species names). Finally, readers may opt to adopt a standardized nomenclatural framework that employs the use of “biovar terms” [3,6], such as “biovar Anthracis”. For example, within the 2020 GSB framework, any anthrax-causing *B. cereus s.l.* strain can be referred to as *B. cereus s.l.* biovar Anthracis or *B. anthracis* [3,6]. Overall, readers can avoid potential taxonomic ambiguities by (i) explicitly and transparently describing the methods and protocols used for taxonomic assignment, and (ii) interpreting taxonomic labels assigned using unknown or unspecified methods with extreme caution.

### 3.3. Whole-Genome Sequencing in Combination with Epidemiological, Microbiological and Clinical Data May Improve Surveillance of Anthrax Cases in the Future

While rare, anthrax-causing *B. cereus s.l.* strains that resemble “*B. cereus*” (per the FDA BAM) represent a serious public health threat, due to the severity of the disease they cause [2,35]. Furthermore, these strains may represent a serious occupational hazard to welders and other metalworkers, as numerous welder anthrax cases among workers have been reported [23,31,35,36,37,56]. It is essential that efforts are undertaken to prepare for and respond to the severe clinical cases caused by these organisms.

To that end, WGS has tremendous potential to improve surveillance of anthrax-causing members of *B. cereus s.l.* [6,9], as it has improved surveillance and source tracking efforts for other pathogens [57,58,59]. Here, WGS confirmed that an anthrax-causing ST78 genome derived from an inhalation anthrax case associated with a welder was effectively identical to an environmental ST78 genome collected from a soil sample taken at the welder’s worksite [35]. These two effectively identical genomes could be distinguished from all publicly available genomes, including those of other anthrax-causing ST78 strains. However, it is essential to note that the link between the environmental and human clinical ST78 genomes sequenced in this study would not have been established without prior epidemiological, clinical, and microbiological efforts, including: (i) linking the clinical case to the worksite associated with the case, and (ii) isolating the organism responsible from a soil sample collected from the worksite [35]. Epidemiological and clinical data (e.g., patient symptoms, patient history), plus microbiological data (e.g., a microbe’s ability to produce a particular toxin, microbial growth temperature), have previously been important for linking *B. cereus s.l.* strains to human illness cases [60]; members of *B. cereus s.l.* are widespread throughout the environment and may potentially be incorrectly linked to a clinical case [1,14,15,60,61]. Hence, it is likely that WGS will be most valuable for elucidating anthrax-causing *B. cereus s.l.* cases and outbreaks when used in combination with epidemiological, clinical, and microbiological data.

Finally, future WGS-based studies of anthrax-causing *B. cereus s.l.* strains that do not belong to the historical *B. anthracis* lineage typically associated with anthrax illness will benefit from increasingly available *B. cereus s.l.* WGS data and associated metadata [6]. For example, international efforts to share and make WGS data publicly available can decrease the amount of time required to solve outbreaks, as well as the public health burdens imposed by the pathogens that cause them [62]. While the amount of publicly available *B. cereus s.l.* genomes is increasing, efforts to sequence *B. cereus s.l.* genomes are lagging far behind other bacterial pathogens (e.g., *Salmonella enterica*, *Listeria monocytogenes*) [63]. Future studies will be able to leverage a greater amount of WGS data and associated metadata to provide further insights into the evolution of anthrax-causing *B. cereus s.l.* and potentially identify novel, emerging lineages prior to human infection.

## 4. Materials and Methods

### 4.1. Clinical Case Information and Environmental Isolate Acquisition

Isolates associated with human clinical cases were acquired as described previously [35]. Additionally, as part of an epidemiologic investigation, samples were collected at both patients’ worksites and homes to identify possible sources of infection [35]. Acquisition of the environmental isolate included in this study (Table 1) was initiated by inoculating 5 g of soil in 15 mL of heart infusion broth (HIB). The soil and HIB were vortexed and sonicated each for 30 s, respectively. This process was repeated 2 additional times. The soil and HIB mixture was then heated in a water bath for 30 min at 65 °C. After allowing the mixture to settle for several minutes, the supernatant and a 1:10 dilution of the supernatant in HIB was plated on R and F Anthracis Chromogenic Agar (R and F Products, Downers Grove, IL, USA). If individual colonies could not be determined from 1:10 dilution plates, additional dilutions were generated and plated. Suspect colonies were isolated for identification by polymerase chain reaction (PCR) and WGS.

### 4.2. Whole-Genome Sequencing

DNA was extracted using the Promega Maxwell 16 and the Maxwell 16 Cell DNA Purification Kit (Promega Corporation, Madison, WI, USA), which was used to generate a draft genome sequence by using the Nextera FLEX Kit (https://www.illumina.com) for library preparation and the iSeq 100 instrument (Illumina) with a 2 × 151-bp kit.

### 4.3. Data Pre-Processing and Quality Control

Illumina paired-end reads associated with each isolate were trimmed and filtered using Trimmomatic v0.39 (Usadel Lab, Düsseldorf, Germany) with the default settings for Illumina paired-end reads [64]. FastQC v0.11.9 (https://www.bioinformatics.babraham.ac.uk/projects/fastqc/, accessed on 23 November 2021; Babraham Bioinformatics, Cambridge, UK) was used to evaluate the quality of the resulting trimmed paired-end reads. For each isolate, trimmed paired-end reads were assembled into contigs using (i) SPAdes v3.15.2 (using the “careful” option; Center for Algorithmic Biology, St. Petersburg, Russia) [65] and (ii) SKESA v2.4.0 [66] (NCBI, Bethesda, MD, USA), and the quality of the resulting assemblies were evaluated using (i) QUAST v5.0.2 (with the “min-contig” option set to 1; Center for Algorithmic Biology, St. Petersburg, Russia) [67] and (ii) CheckM v1.1.3 (via the “lineage_wf” workflow; Australian Centre for Ecogenomics, Brisbane, Australia) [68]. MultiQC v1.11 (Philip Ewels, Stockholm, Sweden) [69] was used to evaluate the quality of all genomic data in aggregate. Contigs produced by SPAdes and SKESA were of similar quality (e.g., for all assemblies, N50 >100.0 Kbp, genome size 5.6–5.7 Mbp, CheckM completeness ≥99.15%, CheckM contamination ≤0.10%; Supplementary Table S1); thus, the contigs produced by SKESA were used in subsequent steps. Prokka v1.13 (Torsten Seemann, Melbourne, Australia) [70] was used to annotate each genome (using the “Bacteria” kingdom database).

### 4.4. Taxonomic Assignment, Sequence Typing, and Detection of Virulence Factors

For each genome, BTyper3 v3.2.0 (Laura Carroll, Heidelberg, Germany) [40] was used to: (i) assign each assembled genome to a taxonomic unit within the 2020 GSB nomenclature [6]; (ii) assign each assembled genome to a *B. cereus s.l.* pseudo-gene flow unit (via “--ani_geneflow True”) [40]; (iii) compare each assembled genome to the genomes of all validly published and effective *B. cereus s.l.* species type strains (*n* = 26, accessed on 20 February 2022) using one-way ANI calculations (via “--ani_typestrains True”); (iv) assign each genome to one of eight *B. cereus s.l.* phylogenetic groups (Group I-VIII), using the sequence of *panC* [40,41,42]; (v) assign each genome to a ST, using the “*B. cereus*” seven-gene MLST scheme implemented in PubMLST (accessed on 24 November 2021; Table 2 and Supplementary Table S1) [38,39]. BTyper3 was additionally used to detect virulence factors in each genome using default thresholds (i.e., using translated nucleotide BLAST, a minimum amino acid identity threshold of 70%, and a minimum coverage threshold of 80%). This step was repeated with the minimum coverage threshold lowered to 0% to confirm that virulence factors discussed in the study were absent from a genome if they were not initially detected using the default threshold (Table 2 and Supplementary Table S1). BTyper3 relied on the following dependencies: FastANI v1.31 (Chirag Jain, Bangalore, India) [18], BLAST v2.9.0+ (NCBI, Bethesda, MD, USA) [71], Biopython v1.79 [72], NumPy v1.18.5 [73], Pandas v1.0.5 [74], and Python v3.8.6.

In addition to comparing the genomes sequenced here to the type strain genomes of all validly published and effective *B. cereus s.l.* species via FastANI (as implemented in BTyper3; see above), ANI values were calculated between the genomes sequenced here and the same *B. cereus s.l.* type strain genomes using (i) OrthoANI (via OAT_cmd.jar v1.40, accessed on 27 April 2021; CJ Bioscience, Seoul, South Korea) [53] and (ii) JSpeciesWS (http://jspecies.ribohost.com/jspeciesws/#analyse, accessed on 20 February 2022; Ribocon, Bremen, Germany) [52]. Additionally, in silico DNA-DNA hybridization (DDH) values were calculated between genomes sequenced here and the same *B. cereus s.l.* type strain genomes using GGDC v3.0 (accessed on 20 February 2022, Table 2; Leibniz Institute DSMZ, Braunschweig, Germany). Results obtained using GGDC Formula 2 (i.e., the formula recommended by GGDC) are reported.

Finally, the Genome Taxonomy Database (GTDB) Release 05-RS95 (17 July 2020) and GTDB-Tk v1.3.0 (i.e., “GTDB R95”) were additionally used to perform taxonomic classification of each genome, using GTDB-Tk’s “classify_wf” workflow and the assembled genome of each isolate as input (Table 2 and Supplementary Table S1; Australian Centre for Ecogenomics, Brisbane, Australia) [47,48,50].

### 4.5. Phylogenomic and Pan-Genomic Comparison to Publicly Available B. mosaicus Genomes

Publicly available *B. cereus s.l.* genomes (*n* = 2890) were downloaded, pre-processed, assembled (where applicable), and annotated as described previously [63] (Supplementary Table S2). Genomes that were assigned to the *B. mosaicus* genomospecies within the 2020 GSB nomenclature were used in subsequent steps unless otherwise specified (*n* = 894 total *B. mosaicus* genomes; Supplementary Table S2). GFF files produced by Prokka were used as input for Panaroo v1.2.8 (Wellcome Sanger Institute, Cambridge, UK) [75], which was used to partition genomic elements into core- and pan-genome components identified among all 894 *B. mosaicus* genomes, using “strict” mode (“--clean-mode strict”), MAFFT as the sequence aligner (“--aligner mafft”), a core genome threshold of 95% (i.e., genes present in ≥95% of genomes were considered to be core genes; “--core_threshold 0.95”), and a protein family sequence identity threshold of 70% (“-f 0.7”, the default). The resulting reference pan-genome coding sequences (CDS) identified by Panaroo underwent functional annotation using the eggNOG-mapper v2 webserver (http://eggnog-mapper.embl.de/, accessed on 8 April 2022; EMBL, Heidelberg, Germany) using default settings [76,77].

The core genome alignment produced by Panaroo was queried using snp-sites v2.5.1 (Wellcome Sanger Institute, Cambridge, UK) [78], which was used to identify core SNPs and constant sites within the nucleotide alignment (using the “-c” and “-C” options, respectively). The resulting core SNPs were supplied as input to IQ-TREE v1.5.4 (IQ-TREE Development Team, http://www.iqtree.org/about/, accessed on 20 July 2022) [79], which was used to construct a maximum likelihood (ML) phylogeny using the following parameters: (i) the general time reversible (GTR) nucleotide substitution model [80], (ii) one thousand replicates of the ultrafast bootstrap approximation [81], and (iii) an ascertainment bias correction to account for the sole use of variant sites (via the “-fconst” option, using constant sites output by snp-sites). All aforementioned steps were performed a second time, with the addition of an outgroup genome (i.e., the genome of *panC* Group IV *B. cereus sensu stricto* species type strain ATCC 14579; NCBI RefSeq Assembly Accession GCF_006094295.1).

All aforementioned steps (i.e., core- and pan-genome analyses, plus ML phylogeny construction with and without an outgroup) were additionally repeated, with the omission of genomes that did not have a year of isolation reported in their associated BioSample [63,82] (Supplementary Table S2). Each resulting phylogeny was rooted and time-scaled using LSD2 v1.4.2.2 (Thu-Hien To, Ås, Norway) [83] and the following parameters: (i) tip dates corresponding to the year of isolation associated with each genome; (ii) constrained mode (-c), with the root estimated using constraints on all branches (-r as); (iii) variances calculated using input branch lengths (-v 1); (iv) 1000 samples for calculating confidence intervals for estimated dates (-f 1000); (v) a sequence length of 5,500,000. For phylogenies that included the outgroup genome, the outgroup was used to root the tree. For phylogenies that did not, the root was estimated using LSD2. Each resulting time-scaled phylogeny, plus the associated orthologous gene cluster presence/absence matrix produced by Panaroo, were supplied as input to Panaroo’s “panaroo-fmg” command, which was used to estimate the pangenome size under the Finite Many Genes (FMG) model [84] with 100 bootstrap replicates.

All aforementioned steps were again repeated, using the following genome sets: (i) genomes assigned to GTDB’s *B. anthracis*, *B. tropicus*, and *B. paranthracis* species (*n* = 607 total genomes, 415 genomes with isolation years); (ii) GTDB’s *B. anthracis* species (*n* = 326 total genomes, 180 genomes with isolation years); (iii) GTDB’s *B. tropicus* species (*n* = 55 total genomes, 46 genomes with isolation years); (iv) the 2020 GSB *B. mosaicus* subspecies *anthracis* lineage (i.e., the clonal, historical *B. anthracis* lineage typically associated with anthrax; *n* = 223 total genomes, 119 with isolation years); (v) genomes that harbored two or more anthrax toxin-encoding genes (i.e., *cya*, *lef*, and/or *pagA*, per BTyper3 default settings, *n* = 176 genomes).

Non-metric multidimensional scaling (NMDS) was performed, using the Panaroo pan-genome orthologous gene cluster presence/absence matrix produced among the 176 anthrax toxin-gene harboring *B. cereus s.l.* genomes as input (see above). Orthologous gene clusters present in all genomes were removed from the alignment, yielding a matrix of 4445 orthologous gene clusters variably present among the 176 genomes. The resulting matrix was supplied as input to the metaMDS command in the vegan v2.5-7 package (Jari Oksanen, Helsinki, Finland) [85] in R v4.1.2 (R Core Team, https://www.r-project.org/, accessed on 20 July 2022) [86], which was used to perform NMDS using a Jaccard distance metric, two dimensions, and a maximum number of 100,000 random starts; a solution was reached in <100 runs. The resulting scores were plotted in R using ggplot2 v3.3.5 (Hadley Wickham, Houston, TX, USA) [87]. The same pan-genome presence/absence matrix was supplied to the anosim and adonis functions in the vegan R package, which were used to conduct analysis of similarity (ANOSIM) [88] and permutational analysis of variance (PERMANOVA) [89] tests, respectively. Each test used lineage membership as a grouping factor (i.e., whether a genome belonged to ST78, the ST365 CC, or the historical *B. anthracis* lineage), a Jaccard distance metric, and 10,000 permutations. Each pairwise lineage combination was additionally tested (eight total tests; raw *p* < 0.001 for all tests, all ANOSIM *R* = 1.0, PERMANOVA *R^2^* = 0.68–0.93). A Bonferroni correction was applied to correct for multiple comparisons.

### 4.6. Anthrax Toxin Gene Presence Ancestral State Reconstruction

The ancestral states of internal nodes within each of the following phylogenies as they related to anthrax toxin gene presence/absence were estimated: (i) the 326-genome GTDB *B. anthracis* phylogeny and (ii) the 55-genome GTDB *B. tropicus* phylogeny. Briefly, the presence or absence of two or more of *cya*, *lef*, and *pagA* within each genome was treated as a binary state, and stochastic character maps were simulated on each phylogeny using the make.simmap function in the phytools v1.0-1 R package (Liam J. Revell, Boston, MA, USA) [90] and the following parameters: (i) the all-rates-different (ARD) model in the ape v5.6-1 R package (Emmanuel Paradis, Montpellier, France); (ii) root node prior probabilities for anthrax toxin gene presence or absence set to either “equal” (i.e., 0.5), or “estimated” (i.e., root node prior probabilities were estimated using the make.simmap function); (iii) 100 or 1000 simulations (for *B. anthracis* and *B. tropicus*, respectively). Results were plotted using the densityMap function in the phytools v1.0-1 R package (Supplementary Figures S3 and S4).

### 4.7. Genomic Comparison to Closely Related B. cereus s.l. Genomes

ANI values were calculated between each of the three genomes sequenced here (Supplementary Table S1), plus all publicly available *B. mosaicus* genomes (per the 2020 GSB framework) using FastANI (Supplementary Table S2). Due to the fact that all ST78 genomes were closely related (ANI >99.8%), core SNPs were identified among all ST78 genomes using the default SNP calling pipeline implemented in Snippy v4.4.0 (Torsten Seemann, Melbourne, Australia) [91], with the closed chromosome of anthrax-causing ST78 *B. cereus s.l.* strain 03BB87 used as a reference (NCBI Nucleotide Accession NZ_CP009941.1). Gubbins v2.4.1 (Wellcome Sanger Institute, Cambridge, UK) [92] was used to remove recombination from the resulting core genome alignment, and snp-sites v2.5.1 [78] was used to query the resulting recombination-free alignment for core SNPs (using the “-c” option). Pairwise core SNP distances between all ST78 genomes were calculated using the “dist.gene” function in the ape v5.6-1 package [93,94] in R v4.1.2 [86]. The resulting core SNPs were additionally supplied as input to IQ-TREE, which was used to construct a ML phylogeny as described above, using the optimal nucleotide substation model selected using ModelFinder [95] (i.e., the K3Pu model) [96]. The resulting phylogeny was viewed in FigTree v1.4.4 (Andrew Rambaut, Edinburgh, Scotland, UK) [97]. All aforementioned steps were repeated, with the omission of the most distantly related ST78 genome (NCBI RefSeq Assembly Accession GCF_002117465.1; Supplementary Figure S2).

Snippy was additionally used to identify SNPs between the two ST78 genomes sequenced here, which were associated with Patient F’s case in Louisiana (i.e., BacLA2020a and BacLA2020b; Table 1). The trimmed paired-end reads associated with the genome of strain BacLA2020a were mapped to the BacLA2020b reference genome; the resulting filtered Variant Call Format (VCF) file produced by Snippy was manually inspected for the presence of SNPs. These steps were repeated, using the trimmed paired-end reads associated with BacLA2020b as input and the BacLA2020a genome as a reference.

## 5. Conclusions

Here, we used WGS to characterize three *B. cereus s.l.* isolates associated with two separate welder anthrax cases that occurred in 2020 among male welders in their thirties in two U.S. Gulf Coast states. All isolates resembled “*B. cereus*” phenotypically (per the FDA BAM). However, by most contemporary metrics used to delineate bacterial species, the isolates associated with each of these cases belonged to separate species (e.g., using the GTDB taxonomy, *B. anthracis* and *B. tropicus*). Furthermore, the isolates differed in terms of the virulence factors they possessed: the ST78 genomes from Louisiana possessed genes encoding the Bps alternative exopolysaccharide capsule, as well as enterotoxin-encoding *hbl* and *cytK-2,* while the ST108 genome from Texas did not. Using all publicly available *B. cereus s.l.* genomes, we predict that members of *B. cereus s.l.* have gained anthrax toxin-encoding genes at least four separate times, specifically: (i) at least once among the historical, clonal *B. anthracis* lineage typically associated with anthrax toxin production; (ii) at least twice within the ST365 CC; and (iii) at least once among ST78. Overall, WGS has the potential to improve surveillance of anthrax-causing *B. cereus s.l.* However, future *B. cereus s.l.* isolation, WGS, and metadata collection efforts will be essential for gaining further insights into the evolution of anthrax-causing *B. cereus s.l.* in the U.S. and around the world.

## Figures and Tables

**Table 1:** *Bacillus cereus sensu lato* (*B. cereus s.l.*) genomes sequenced in this study and their corresponding metadata (*n* = 3).

Isolate	Year of Isolation	Geographic Location of Isolation	Isolation Source (General)	Isolation Source (Details)	Host Illness	Host Age (Years)	Host Sex	Host Occupation	Host Outcome
BacLA2020a	2020	Louisiana, USA	Human clinical case	Patient F ^a,b^	Severe anthrax pneumonia	39	Male	Welder	Recovered ^c^
BacLA2020b	2020	Louisiana, USA	Environmental isolate	Soil sample from Patient F’s worksite ^d^	NA ^e^	NA ^e^	NA ^e^	NA ^e^	NA ^e^
BacTX2020a	2020	Texas, USA	Human clinical case	Patient G ^a^	Severe anthrax pneumonia	34	Male	Welder	Fatal

^a^ Data from human clinical cases described by Dawson et al. [35]; ^b^ Patient F is a Mississippi resident who had recently worked as a welder in Louisiana [35]; ^c^ Patient F recovered after receiving anthrax antitoxin [35]; ^d^ Isolate BacLA2020b was recovered from a soil sample taken at Patient F’s worksite in Louisiana via an investigation conducted by the U.S. Centers for Disease Control and Prevention (CDC) [35]; ^e^ NA, not applicable for environmental isolates.

**Table 2:** Taxonomic assignment of *B. cereus s.l.* genomes sequenced in this study (*n* = 3).

	Single- and Multi-Locus Sequence Typing			Toxin Genes	Capsule Genes ^d^			Whole-Genome-Based Taxonomic Assignment		
Genome	*panC* Group ^a^	MLST ST ^b^	*rpoB* AT ^c^	Anthrax Toxin Genes ^d,e^	Cap ^f^	Bps ^g^	Has ^h^	Closest Species Type Strain (ANI; DDH) ^i^	GTDB Species ^j^	2020 GSB Taxonomy ^k^
BacLA2020a	II	78	365	+	−	+	+	*B. tropicus* (96.5%; 69.7%)	*B. tropicus*	*B. mosaicus* biovar Anthracis; *B. anthracis*
BacLA2020b	II	78	365	+	−	+	+	*B. tropicus* (96.5%; 69.7%)	*B. tropicus*	*B. mosaicus* biovar Anthracis; *B. anthracis*
BacTX2020a	III	108	120	+	−	−	+	*B. anthracis* (97.4%; 76.1%)	*B. anthracis*	*B. mosaicus* biovar Anthracis; *B. anthracis*

^a^*panC* phylogenetic group (I-VIII) assigned using BTyper3 v3.2.0 [40]; all genomes were assigned to Group III using the seven-group scheme developed by Guinebretiere et al. (https://www.tools.symprevius.org/bcereus/; accessed on 20 February 2022) [41,42]; ^b^ Sequence type (ST) assigned using PubMLST’s seven-gene multi-locus sequence typing (MLST) scheme for “*B. cereus*” [38,39] and BTyper3 v3.2.0 [40]; ^c^ *rpoB* allelic type (AT) [43] assigned using the original BTyper (BTyper v2.3.4) [44]; ^d^ Selected virulence factors detected in each genome using BTyper3 v3.2.0 [40]; presence and absence of virulence factors are denoted by “+” and “−“, respectively. Virulence factors were first detected using default thresholds (70% amino acid identity and 80% coverage); virulence factor absence was confirmed by lowering the minimum coverage threshold to 0%; ^e^ Each of anthrax toxin-encoding *cya*, *lef*, and *pagA* were detected in all three genomes sequenced here; ^f^ None of poly-γ-D-glutamic acid (polyglutamate Cap) capsule-encoding *capBCADE* were detected in any genome sequenced here; ^g^ All of exopolysaccharide (Bps) capsule-encoding *bpsXABCDEFGH* were detected in BacLA2020a and BacLA2020b; one *bpsE*-like hit was detected in BacTX2020a (79% amino acid identity, 95% coverage); ^h^ All of hyaluronic acid (Has) capsule-encoding *hasABC* were detected in all three genomes sequenced here; ^i^ Closest species type strain genome relative to all validly published and effective *B. cereus s.l.* species (*n* = 26; accessed on 20 February 2022) via average nucleotide identity (ANI) and in silico DNA-DNA hybridization (DDH). ANI values were calculated using FastANI v1.31 [18] and BTyper3 v3.2.0 [40]; DDH values were calculated using the Genome-to-Genome Distance Calculator v3.0 (accessed on 20 February 2022) [45,46]; ^j^ Genome Taxonomy Database (GTDB) species assigned using GTDB Release 05-RS95 (17 July 2020) and GTDB-Tk v1.3.0 [47,48]; ^k^ Species and biovars assigned using the 2020 Genomospecies-Subspecies-Biovar (GSB) nomenclatural framework for *B. cereus s.l.* [6] and BTyper3 v3.2.0 [40]; multiple taxonomic labels are listed (separated by a semi-colon), as all genomes sequenced here can be referenced using shorted biovar notation.

## Data Availability

NCBI accession numbers for all genomes used in this study are available in Supplementary Tables S1–S3. Genomes sequenced in this study are available under NCBI BioProject Accession PRJNA849252.

## Supplementary Materials

The following supporting information can be downloaded at: https://www.mdpi.com/article/10.3390/pathogens11080856/s1, Supplementary Figure S1: Maximum likelihood phylogeny constructed using core genes detected among 894 genomes assigned to the *B. mosaicus* genomospecies; Supplementary Figure S2: Maximum likelihood phylogenies constructed using core SNPs identified among (A) all PubMLST ST78 genomes (*n* = 8 genomes) and (B) the same set of ST78 genomes, with the most distantly related genome removed; Supplementary Figure S3: Maximum likelihood phylogeny constructed using core SNPs detected among 55 genomes assigned to the Genome Taxonomy Database (GTDB) *B. tropicus* species; Supplementary Figure S4: Maximum likelihood phylogeny constructed using core SNPs detected among 326 genomes assigned to the Genome Taxonomy Database (GTDB) *B. anthracis* species; Supplementary Table S1: Genomes sequenced in this study (*n* = 3); Supplementary Table S2: Publicly available genomes used in this study (*n* = 2887); Supplementary Table S3: Publicly available anthrax toxin gene-harboring genomes, which do not belong to the clonal, historical *B. anthracis* lineage typically associated with anthrax; Supplementary Table S4: Orthologous gene clusters found exclusively in anthrax toxin gene-harboring members of the clonal, historical *B. anthracis* lineage (i.e., *B. mosaicus* subsp. *anthracis* biovar Anthracis within the 2020 *B. cereus s.l.* Genomospecies-Subspecies-Biovar taxonomy) relative to all other anthrax toxin gene-harboring genomes; Supplementary Table S5: Orthologous gene clusters found exclusively in anthrax toxin gene-harboring PubMLST Sequence Type 78 (ST78) genomes relative to all other anthrax toxin gene-harboring genomes; Supplementary Table S6: Orthologous gene clusters found exclusively in anthrax toxin gene-harboring PubMLST Sequence Type 365 Clonal Complex (ST365 CC) genomes relative to all other anthrax toxin gene-harboring genomes; Supplementary Table S7: Orthologous gene clusters exclusively absent from anthrax toxin gene-harboring members of the clonal, historical *B. anthracis* lineage (i.e., *B. mosaicus* subsp. *anthracis* biovar Anthracis within the 2020 *B. cereus s.l.* Genomospecies-Subspecies-Biovar taxonomy) relative to all other anthrax toxin gene-harboring genomes; Supplementary Table S8: Orthologous gene clusters exclusively absent from anthrax toxin gene-harboring PubMLST Sequence Type 78 (ST78) genomes relative to all other anthrax toxin gene-harboring genomes; Supplementary Table S9: Orthologous gene clusters exclusively absent from anthrax toxin gene-harboring PubMLST Sequence Type 365 Clonal Complex (ST365 CC) genomes relative to all other anthrax toxin gene-harboring genomes; Supplementary Table S10: Orthologous gene clusters exclusively present in anthrax toxin gene-harboring PubMLST Sequence Type 78 (ST78) genomes relative to all other *B. mosaicus* genomes (per the 2020 *B. cereus s.l.* Genomospecies-Subspecies-Biovar taxonomy), plus the *panC* Group IV outgroup genome.
