# Supplementary material for: Strains Associated with Two 2020 Welder Anthrax Cases in the United States Belong to Separate Lineages within Bacillus cereus sensu lato
Source: Pathogens. 2022 Jul 29;11(8):856. doi: 10.3390/pathogens11080856 (PMC9413466; doi:10.3390/pathogens11080856)
Supplement: Supplementary file 1 [file pathogens-11-00856-s001.zip › SupplementaryFigureS3_GTDBtropicus_ancestral_state.pdf]

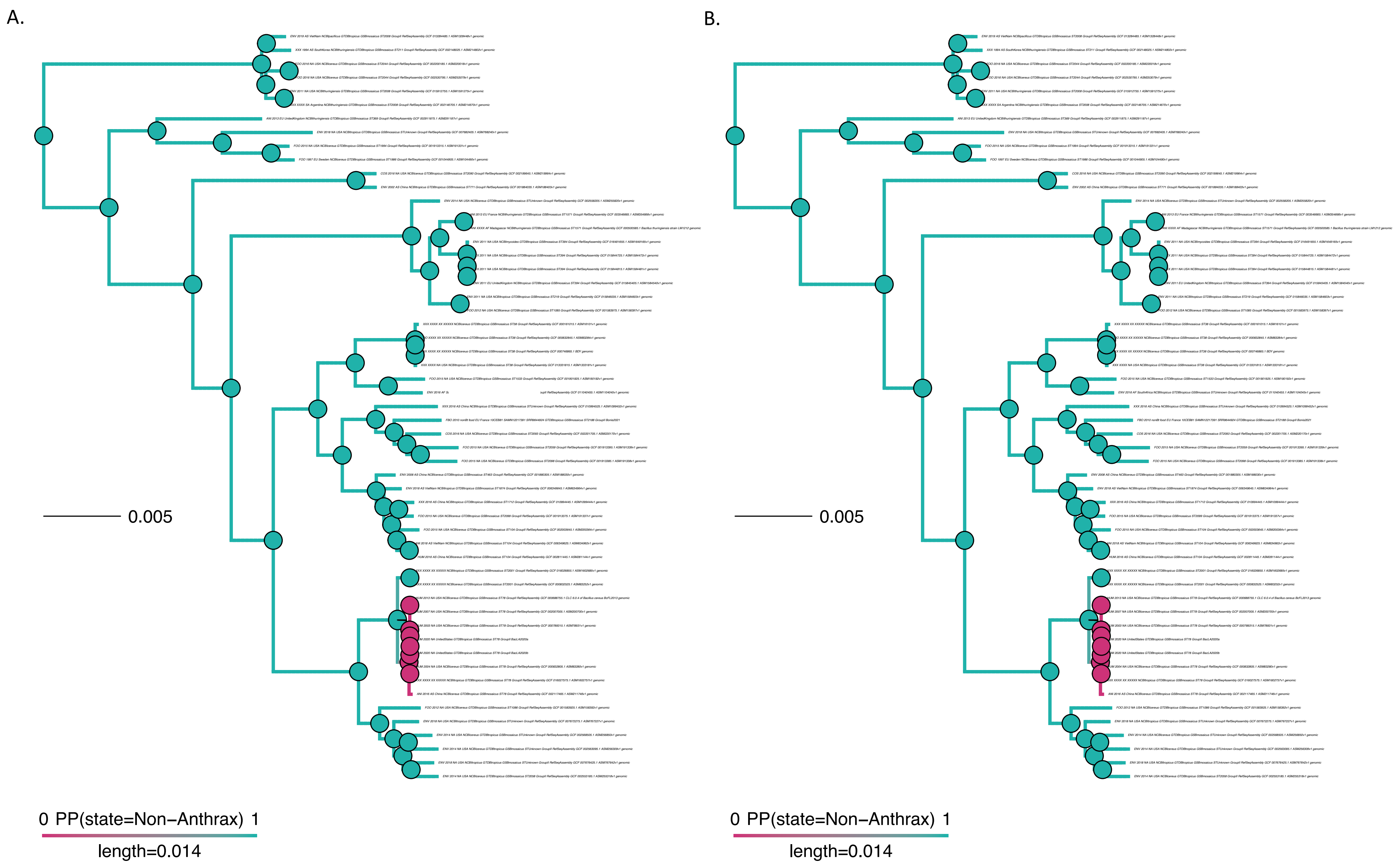

Supplementary Figure S3. Maximum likelihood phylogeny constructed using core SNPs detected among 55 genomes assigned to the Genome Taxonomy Database (GTDB) *B. tropicus* species, plus GTDB *B. paranthracis* outgroup genome *B. cereus s.l.* strain AH187 (NCBI RefSeq Assembly Accession GCF_000021225.1; omitted for readability). Tree edge and node colors correspond to the posterior probability (PP) of anthrax toxin gene absence (i.e., “Non-Anthrax”), obtained using an empirical Bayes approach, in which a continuous-time reversible Markov model was fitted, followed by 1,000 simulations of stochastic character histories using the fitted model and tree tip states. Root node prior probabilities for “Anthrax” and “Non-Anthrax” states were (A) equal (i.e., 0.5 each) or (B) estimated using the make.simmap function in the phytools package in R. Trees are rooted using the outgroup genome, and branch lengths are reported in substitutions per site.
