# Supplementary material for: Strains Associated with Two 2020 Welder Anthrax Cases in the United States Belong to Separate Lineages within Bacillus cereus sensu lato
Source: Pathogens. 2022 Jul 29;11(8):856. doi: 10.3390/pathogens11080856 (PMC9413466; doi:10.3390/pathogens11080856)
Supplement: Supplementary file 1 [file pathogens-11-00856-s001.zip › SupplementaryFigureS1_GSBmosaicus.pdf]

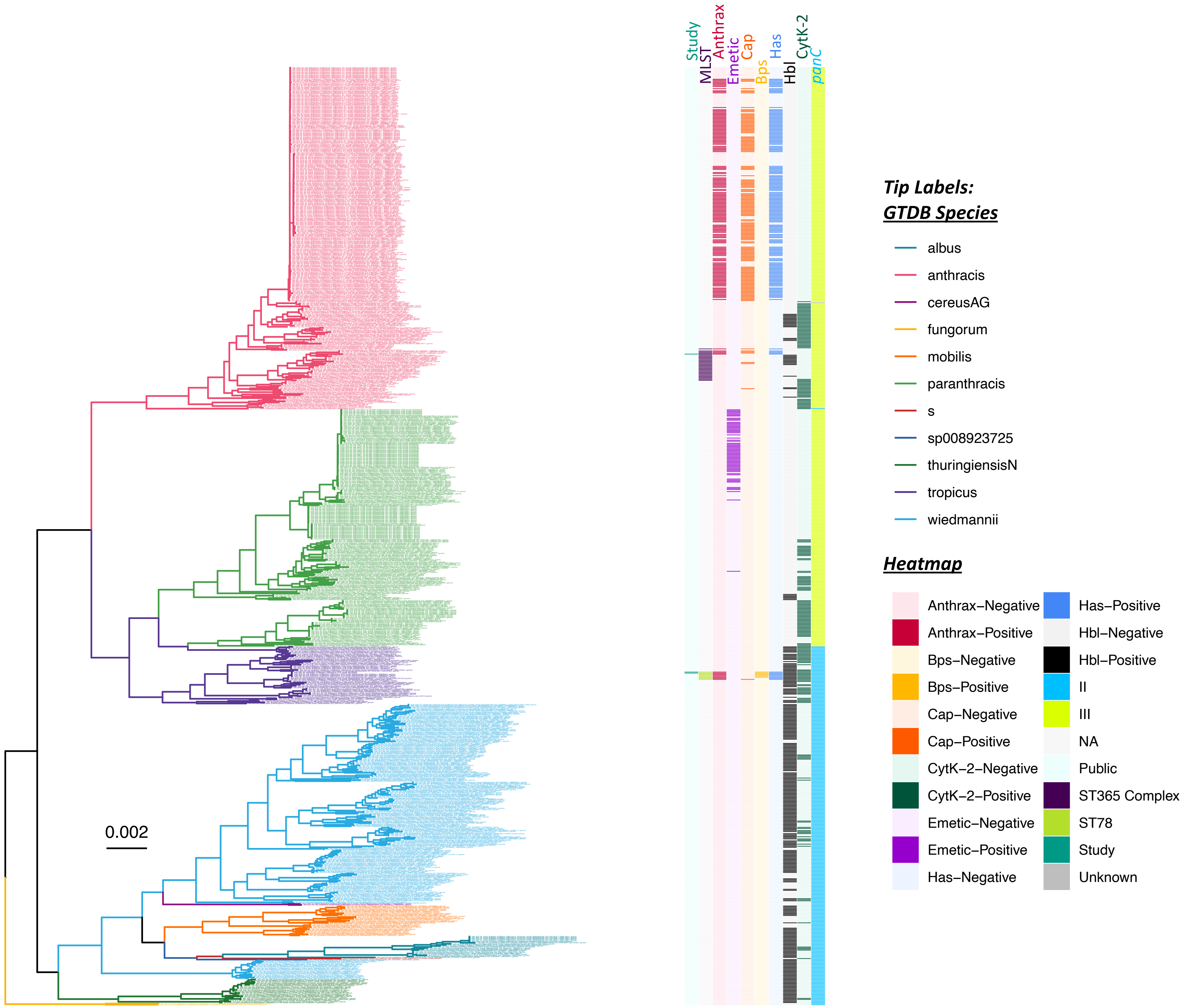

Supplementary Figure S1. Maximum likelihood phylogeny constructed using core genes detected among 894 genomes assigned to the *B. mosaicus* genomospecies within the 2020 *B. cereus s.l.* Genomospecies-Subspecies-Biovar (GSB) taxonomy, plus *panC* Group IV outgroup genome *B. cereus sensu stricto* strain ATCC 14579 (NCBI RefSeq Assembly Accession GCF_006094295.1; omitted for readability). Tip label colors denote Genome Taxonomy Database (GTDB) species assignments. The heatmap to the right of the phylogeny denotes the following (from left to right): (i) whether a genome was sequenced in this study or publicly available ("Study"); (ii) selected PubMLST lineages assigned using seven-gene multi-locus sequence typing, to which the three genomes sequenced in this study were assigned ("MLST"); (iii) whether a genome possessed two or more anthrax toxin-encoding genes (*cya, lef, pagA*) or not ("Anthrax"); (iv) whether a genome possessed three or more cereulide synthetase (emetic toxin)-encoding genes (*cesABCD*) or not ("Emetic"); (v) whether a genome possessed four or more polyglutamate capsule-encoding genes (*capBCADE*) or not ("Cap"); (vi) whether a genome possessed six or more Bps exopolysaccharide-encoding genes (*bpsXABCDEFGH*) or not ("Bps"); (vii) whether a genome possessed two or more hyaluronic acid capsule-encoding genes (*hasABC*) or not ("Has"); (viii) whether a genome possessed three or more hemolysin BL diarrheal enterotoxin-encoding genes (*hblABCD*) or not ("Hbl"); (ix) whether a genome possessed cytotoxin K-encoding *cytK-2* or not ("CytK-2"); (x) the *panC* Group to which each genome was assigned (using BTyper3 and an eight-group scheme; "*panC*"). The phylogeny was rooted along the outgroup genome, with branch lengths reported in substitutions per site.
