# Supplementary material for: Strains Associated with Two 2020 Welder Anthrax Cases in the United States Belong to Separate Lineages within Bacillus cereus sensu lato
Source: Pathogens. 2022 Jul 29;11(8):856. doi: 10.3390/pathogens11080856 (PMC9413466; doi:10.3390/pathogens11080856)
Supplement: Supplementary file 1 [file pathogens-11-00856-s001.zip › SupplementaryFigureS2_ST78.pdf]

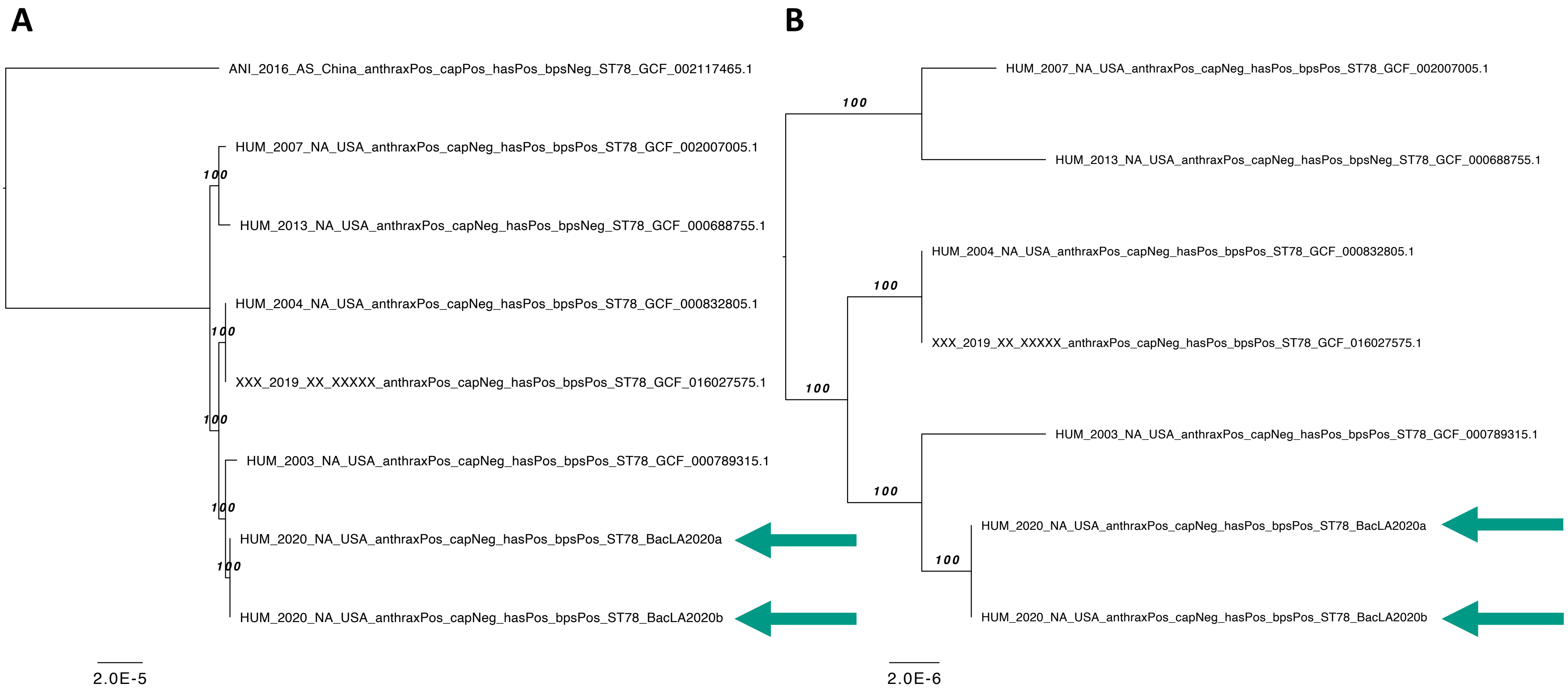

Supplementary Figure S2. Maximum likelihood phylogenies constructed using core SNPs identified among (A) all PubMLST ST78 genomes (*n* = 8 genomes) and (B) the same set of ST78 genomes, with the most distantly related genome removed (i.e., NCBI RefSeq Assembly Accession GCF_002117465.1; *n* = 7 genomes). Tip labels corresponding to the two ST78 genomes sequenced in this study are annotated with green arrows. Branch labels correspond to branch support percentages produced using one thousand replicates of the ultrafast bootstrap approximation. Phylogenies are rooted at their respective midpoints, with branch lengths reported in substitutions per site. The core genome alignment for each phylogeny was produced using Snippy, and Gubbins was used to remove recombination from each alignment. Core SNPs were extracted from each recombination-free alignment using snp-sites, and the resulting core SNPs were suppled as input to IQ-TREE, which was used to construct each phylogeny.
